# Effects of astaxanthin on VEGF level and antioxidation in human aqueous humor: difference by sex

**DOI:** 10.3164/jcbn.18-110

**Published:** 2019-04-18

**Authors:** Hirotaka Hashimoto, Kiyomi Arai, Jiro Takahashi, Makoto Chikuda

**Affiliations:** 1 Tsukuba Hashimoto Optical Clinic, 530 Furuku, Tsukuba-shi, Ibaraki 305-0021, Japan; 2 Department of Ophthalmology, Saitama Medical Center, Dokkyo Medical University, 2-1-50 Minamikoshigaya, Koshigaya-shi, Saitama 343-8555, Japan; 3 Fuji Chemical Industry Co., Ltd., 55 Yokohoonji, Kamiichi-machi, Nakaniikawa-gun, Toyama 930-0397, Japan

**Keywords:** astaxanthin, aqueous humor, superoxide, oxidation, VEGF

## Abstract

In our previous report, we showed the effect of astaxanthin intake on VEGF level in the aqueous humor and the relationship between VEGF level and reactive oxygen species-related parameters and other relevant factors. VEGF level is associated with total hydroperoxide level, and a multivariate analysis identified sex as a secondary factor affecting these relationships. Here, we analyzed the effects of astaxanthin on the relationship between VEGF level and reactive oxygen species-related parameters by sex. Patients (16 males and 19 females, aged 71.3 and 70.6, respectively) underwent bilateral cataract surgery on one side before and the other side after astaxanthin treatment (6 mg/day for 2 weeks). Levels of VEGF, hydrogen peroxide, and total hydroperoxide, and O_2_^•−^ scavenging activity, were measured in the aqueous humor. In females only, VEGF level was negatively correlated with O_2_^•−^ scavenging activity before the astaxanthin intake (*r* = −0.6, *p*<0.01) and positively correlated with total hydroperoxide level before and after the astaxanthin intake (*r* = 0.7 and 0.8, respectively, *p*<0.01). In conclusion, astaxanthin appears to affect O_2_^•−^ scavenging activity in the aqueous humor in females, and is likely to be involved in the control of VEGF levels in the anterior eye.

## Introduction

The role of oxidation in the development or aggravation of various pathologies including cancer or inflammation as well as aging has recently been determined, and research activities on antioxidative substances have subsequently increased. In the field of ophthalmology, this interest is particularly apparent in research related to cataracts, diabetic retinopathy, uveitis, and age-related macular degeneration (AMD).^(1)^ This has resulted, for example, in the clinical use of lutein, a type of carotenoid, as a supplement to help prevent the development of AMD.^(2)^

**Fig. 1:**
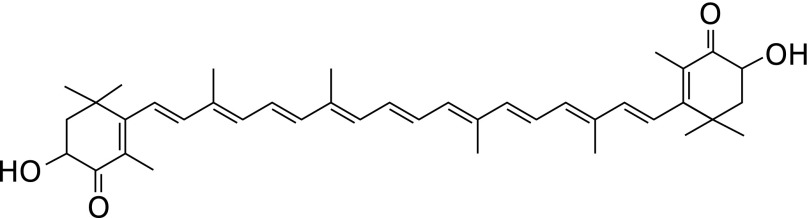
Structural formula of astaxanthin.

Astaxanthin (AX, Fig. 1), another carotenoid, has also garnered research interest, and evidence of its strong antioxidative effects and safety has been disseminated in multiple research areas.^(3,4)^ We have previously reported the effects of AX supplementation in ophthalmology, including the suppression of inflammation after cataract surgery and changes in the levels of oxidation-related parameters including superoxide (O_2_^•−^) scavenging activity, hydrogen peroxide (H_2_O_2_), and total hydroperoxides (TH) in aqueous humor, and have analyzed the correlations between these parameters.^(5–9)^

Meanwhile, antioxidative substances and intravitreous injection of inhibitors of vascular endothelial growth factor (VEGF), a signaling protein that contributes to this pathology via angiogenesis and the enhancement of vascular permeability, are widely used to prevent the development or progression of wet-type AMD.^(10,11)^ Our study group previously evaluated the effect of AX on VEGF levels as well as on oxidation status in the aqueous humor, and analyzed the relationships between the levels of VEGF and oxidation-related parameters (O_2_^•−^, H_2_O_2_, and TH).^(12)^ We further performed multivariate analysis on the contributions of factors such as sex, age, and diabetic status of patients to the levels of VEGF in the aqueous humor and to the change in VEGF levels following AX intake. Our findings were: (i) that the primary factor affecting VEGF levels in the aqueous humor before and after the AX intake is TH, and that the levels of VEGF in the aqueous humor and TH correlated with each other; and (ii) that a secondary factor affecting VEGF levels in the aqueous humor before AX intake is superoxide (O_2_^•−^) scavenging activity, and that VEGF levels in the aqueous humor after AX intake are affected by sex.

In the present study, with a focus on our previous finding that sex is a secondary factor affecting aqueous VEGF level in the aqueous humor after AX intake, we analyzed sex differences in the effect of AX intake on VEGF levels in the aqueous humor and on the relationships between VEGF levels and oxidation-related parameters.

## Patients and Methods

Thirty-five patients, including 16 males (71.3 ± 6.4 years) and 19 females (70.6 ± 7.4 years), who underwent bilateral cataract surgery (intraocular lens implantation) at Tsukuba Hashimoto Optical Clinic were enrolled after providing informed consent. Patients with inflammatory conditions such as uveitis with a high degree of refractive error (8.0 diopters or above) and patients taking other supplements were excluded. The protocol was approved by the Bioethics Committee, Dokkyo Medical University Saitama Medical Center (approval number: 22025) and the study was performed in accordance with the ethical standards of the 1964 Declaration of Helsinki and its later amendments.

Patients initiated AX treatment (6 mg/day) immediately after undergoing surgery in one eye, and subsequently underwent surgery in the other eye 2 weeks later. The AX supplement used in this study was Astavita (Fuji Chemical Industry, Toyama, Japan), derived from algae. Aqueous humor was collected from each of eye during surgery for analysis of oxidation-related parameters (O_2_^•−^scavenging activity and H_2_O_2_ and TH level) and VEGF level.^(12–17)^ The relationships between these parameters and VEGF level were analyzed according to sex before and after AX intake. Correlations between parameters were analyzed using Pearson’s correlation coefficient, and quantitative changes before and after AX intake were analyzed using Wilcoxon’s signed rank sum test. Values of *p*<0.05 were considered statistically significant.

Nitro blue tetrazolium (NBT) reduction was used to measure O_2_^•−^ scavenging activity and a titanium colorimetric method was used to measure H_2_O_2_.^(13,14)^ The NBT assay was performed using a SOD Test kit, “SOD Test Wako R” (Wako Pure Chemical Industries Ltd., Osaka, Japan). This method measures general O_2_^•−^scavenging activities using scavengers other than superoxide dismutase (SOD), including reduced glutathione (GSH) and l-ascorbic acid (l-AsA).

TH was measured by microassay using a Free d-ROMs fast reagent (Diacron Srl, Grosseto, Italy).^(15)^ *N*,*N*-diethylparaphenylenediamine, the chromogen pigment in the Free d-ROMs reagent, reacts with H_2_O_2_, lipid peroxides, peroxidized nucleic acids, and nucleotides, as well as peroxides of proteins, peptides, and amino acids. Thus, measured TH levels indicate the total amount of peroxidized (-OOH modified) substances.^(16)^

VEGF levels were measured with an enzyme-linked immunosorbent assay (ELISA) using Quantikine (R&D Systems, Minneapolis, MN), which detects VEGF_165_ with a limit of detection of 5.0 pg/ml.^(17)^[Table T1]

## Results

### VEGF levels in the aqueous humor before and after the AX intake

**Fig. 2:**
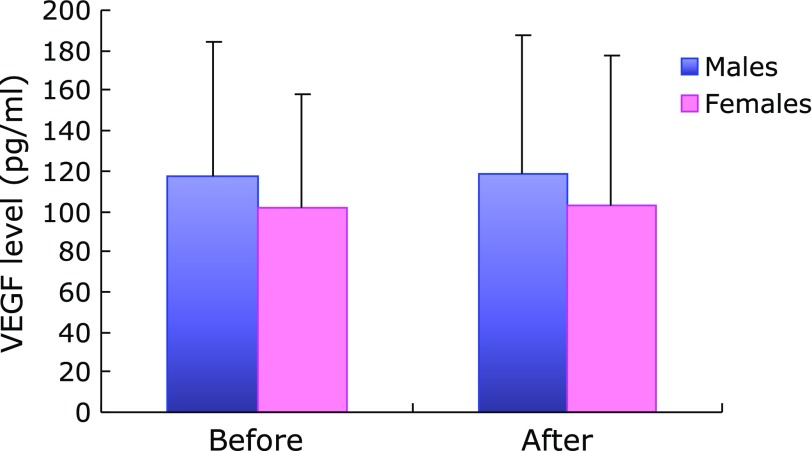
Changes in parameters in aqueous humor before and after AX intake.

**Fig. 3:**
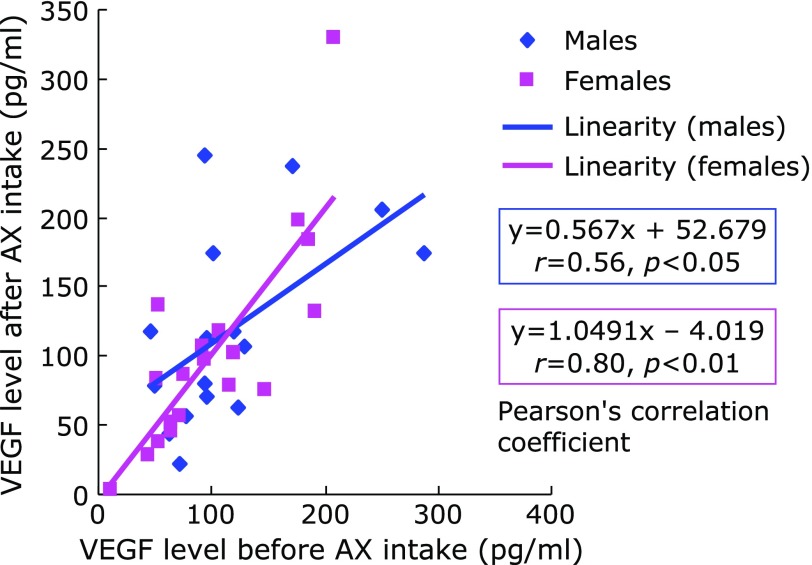
VEGF levels in aqueous humor before and after AX intake.

There was no significant difference in VEGF levels before and after AX administration in either males or females (Fig. 2). Significant positive correlations were observed between VEGF levels before and after AX intake in both males and females (*r* = 0.56, *p*<0.05 for males; *r* = 0.80, *p*<0.01 for females) (Fig. 3).

### Effects of AX intake on the relationship between VEGF levels and various factors in aqueous humor

**Fig. 4:**
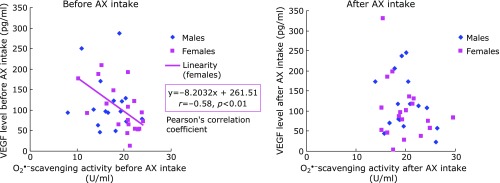
Relationship between superoxide scavenging activity and VEGF levels in aqueous humor.

**Fig. 5:**
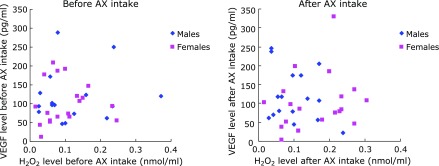
Relationship between H_2_O_2_ and VEGF levels in aqueous humor.

**Fig. 6:**
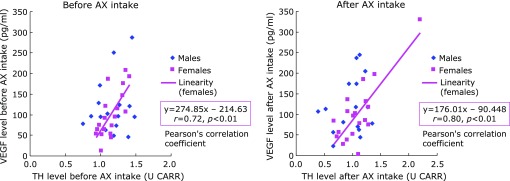
Relationship between TH and VEGF levels in aqueous humor.

For the relationship between VEGF level and O_2_^•−^ scavenging activity, a negative correlation (*r* = –0.58, *p*<0.01) observed only in females prior to AX intake did not persist after AX intake. No correlation was seen in males, either before or after AX intake (Fig. 4). For the relationship between VEGF level and H_2_O_2_ level, no correlation was seen for either males or females before and after AX intake (Fig. 5). For the relationship between VEGF and TH level, a positive correlation (*r* = 0.72, *p*<0.01) observed only in females prior to AX intake was maintained after AX intake (*r* = 0.80, *p*<0.01) (Fig. 6).

## Discussion

In this study, no significant quantitative change in VEGF levels was observed in the aqueous humor before and after AX administration in male or female patients. Extreme changes in VEGF levels are unfavorable since VEGF is constantly required in the body and significant decreases for any reason may affect the maintenance of homeostasis, for example, in cardiac muscle or brain. The amount of AX received by each patient in this study (6 mg) was equivalent to that contained in 300 g of salmon fillet consumed as a meal. A study conducted by another institution, which orally administered AX (8 mg/day) for 8 weeks, showed significantly larger increase in blood AX level than the placebo group.^(3)^ In our study, it is surmised that blood AX levels had increased after the AX intake, we did not observe any quantitative change in the VEGF level in the aqueous humor between before and after AX administration in either sexes. Thus, it was confirmed that this amount of AX (6 mg/day) does not significantly affect the VEGF level in the aqueous humor. It has previously been reported that VEGF levels in the retinal pigment epithelium (RPE) are suppressed by AX administration in an experimental mouse model of macular degeneration induced by laser irradiation.^(18)^ In the present study, AX intake did not lead to a significant decrease in VEGF level, even though specimens were also obtained from inside the eye. The specimen was aqueous humor, which crosses the blood–aqueous barrier, potentially explaining this finding; furthermore, the dose of AX given to patients in this study was markedly lower than that given to mice (up to 100 mg/kg body weight).

In females, O_2_^•−^ scavenging activity prior to AX administration negatively correlated with VEGF level. We considered this to indicate that O_2_^•−^ generation is involved in the increase in VEGF level in the aqueous humor. Although AX is known to be water insoluble in its unbound form, it becomes water soluble when bound to proteins and is transported by lipoproteins in the bloodstream. Water-soluble protein-bound AX is reported to have strong O_2_^•−^ scavenging activity.^(19,20)^ Thus, there is a possibility that AX administration can contribute to decreased of O_2_^•−^ in the aqueous humor. Furthermore, in non-aqueous environments, AX is reported to show O_2_^•−^ scavenging activity, albeit weakly.^(21)^ The negative correlation between VEGF and O_2_^•−^ scavenging activity observed in females before AX administration was absent after AX administration, likely because of the modifying effect of AX on O_2_^•−^ scavenging activity. Meanwhile, estrogen, a female hormone, is known to exert antioxidative effects, including O_2_^•−^ scavenging activity and suppression of lipid peroxidation.^(22,23)^ A relationship between decreased estrogen level in postmenopausal women and various pathologies including cardiovascular diseases, involving advanced oxidation reactions, has been established.^(24)^ We inferred that lower levels of female hormone having antioxidative capacity in postmenopausal female patients might explain why AX intake had a greater effect on the correlation between VEGF level O_2_^•−^ scavenging activity in female patients.

In this study, the levels of H_2_O_2_, a type of reactive oxygen species generated from O_2_^•−^ scavenging, tended to be higher in the aqueous humor of females after AX intake, although no significant correlation was observed between VEGF levels and H_2_O_2_ in the aqueous humor for either sex. VEGF may therefore be involved in the generation or removal of H_2_O_2_ to a very limited extent.

A positive correlation was observed between VEGF level and TH level both before and after AX intake, but in females only. Furthermore, TH level significantly decreased following AX intake in females. As described in the Patients and Methods section, TH is a general indicator of the amount of various peroxides, including H_2_O_2_, lipid peroxides, peroxides of nucleic acids and nucleotides, and peroxides of proteins, peptides, and amino acids, and represents the overall degree of oxidation and oxidative stresses. A positive correlation between VEGF and TH levels observed in this study indicates the close relationship between VEGF levels and oxidative stress in the aqueous humor of females. We considered this correlation to be maintained through AX intake as TH is a general peroxide indicator, distinct from O_2_^•−^ scavenging activity, which showed a change in correlation with VEGF level after AX intake.

In males, no significant change in VEGF level before and after AX intake was observed, and none of the oxidation-related parameters measured in this study showed a significant correlation with VEGF either before or after AX administration. AX intake was therefore considered to have little effect on VEGF levels in the aqueous humor of males.

In conclusion, VEGF levels in the aqueous humor of females appear to be regulated by the overall extent of oxidation. Furthermore, AX appears to affect O_2_^•−^ scavenging in the aqueous humor in females and is also likely to be involved in regulating VEGF levels in the anterior segment of the eye.

## Figures and Tables

**Table 1 T1:** Identification of oxidation-related parameters affecting VEGF levels in aqueous humor by stepwise multiple regression analyses before and after astaxanthin (AX) intake

Before AX intake, factors affecting VEGF level in aqueous humor		
Rank	Item	Standardized partial regression coefficient (β)
1	TH level	0.3339
2	O_2_^•−^ scavenging activity	–0.271

Multiple regression equation: (VEGF before AX intake) = 115.3557 × (TH before AX intake) – 4.0149 × (O_2_^•−^ scavenging activity before AX intake) + 48.5403		
Multiple correlation coefficient: *r* = 0.49 (*p* = 0.0123)		
TH, total hydroperoxide.		

After AX intake, factors affecting VEGF level in aqueous humor		
Rank	Item	Standardized partial regression coefficient (β)
1	TH level	0.6026
2	gender	0.2381

Multiple regression equation: (VEGF after AX intake) = 136.5143 × (TH after AX intake) + 33.5714 × (gender) + 47.1114		
Multiple correlation coefficient: *r* = 0.60 (*p* = 0.0008)		
TH, total hydroperoxide.		

## Abbreviations

AMD: age-related macular degeneration
AX: astaxanthin
ELISA: enzyme-linked immunosorbent assay
GSH: glutathione
H_2_O_2_: hydrogen peroxide
l-AsA: l-ascorbic acid
NBT: nitro blue tetrazolium
O_2_^•−^: superoxide
-OOH: peroxidated substances
RPE: retinal pigment epithelium
SOD: superoxide dismutase
TH: total hydroperoxide
VEGF: vascular endothelial growth factor
